# Order and rack sequencing with a rack buffer area and order due dates in robotic mobile fulfillment systems

**DOI:** 10.1371/journal.pone.0359529

**Published:** 2026-10-06

**Authors:** Yi Yang, Ruoxuan Huang, Dongping Liu, Qinghong Shuai

**Affiliations:** 1 School of Management Science and Engineering, Southwestern University of Finance and Economics, Chengdu, Sichuan, China; 2 School of Finance, Southwestern University of Finance and Economics, Chengdu, Sichuan, China; Chang Gung University, TAIWAN

## Abstract

With the increasing requirements for fulfillment timeliness in e-commerce and the wide application of robotic mobile fulfillment systems, the order picking process needs to balance rack reuse efficiency and on-time order completion. This paper studies an order and rack sequencing problem with a rack buffer area under order due dates. Given limited rack buffer capacity, known order requirements, and order due dates, we jointly optimize the order entry sequence, rack service sequence, and buffer retention decisions. To minimize rack movements and total weighted order tardiness, we formulate a mixed-integer programming model and develop a deadline-aware interactive heuristic with neighborhood search (DA-IHNS). The performance of the proposed model and algorithm is evaluated through randomly generated instances and comparative experiments. In the exact-solution validation, DA-IHNS matches the CPLEX objective on all instances with *N* = 4 and 6, while the mean relative difference is 3.33% for *N* = 8. For the main test instances with *N* = 30, 50, and 80, DA-IHNS achieves mean paired objective reductions of 83.65%–93.98% relative to IH and 79.86%–90.26% relative to EDD-IH, the two rule-based benchmarks. Across the tested synthetic settings, the mean advantage of DA-IHNS over the two rule-based benchmarks remains positive under all examined due-date tightness levels, while buffer capacity and the tardiness penalty coefficient affect the balance between rack reuse and order timeliness. This paper provides a reference for rack buffer configuration, order and rack sequencing, and tardiness control in e-commerce warehousing.

## 1. Introduction

In recent years, the rapid development of e-commerce and instant delivery services has placed increasing operational pressure on warehousing systems, including large order volumes, diversified product categories, and short response times. Order picking, as one of the most labor-intensive and time-consuming warehouse operations, directly affects order fulfillment efficiency and customer service levels [1–3]. In traditional picker-to-parts picking systems, pickers need to move frequently among storage racks, and operational efficiency is easily affected by walking distance, storage layout, and order structure. To improve warehouse operating efficiency, robotic mobile fulfillment systems (RMFSs) have been increasingly adopted in e-commerce warehousing. In such systems, robots transport the required racks to picking stations, thereby realizing a parts-to-picker picking mode, reducing manual walking time, and improving the level of automation [4–7].

In RMFSs, order processing and rack service are closely interdependent. Orders processed concurrently may share racks, reducing repeated rack retrievals from storage, whereas poorly coordinated order and rack sequences can increase handling requirements. This interdependence has been documented in parts-to-picker and RMFS operations [8–10].

A finite rack buffer creates additional opportunities for rack reuse by allowing selected racks to remain near the picking station instead of returning immediately to storage. Han et al. [11] jointly consider order sequencing, rack retrieval sequencing, and rack buffering and show that temporary rack retention can reduce repeated retrievals. Because buffer capacity is limited, retention decisions must account for the future reuse value of competing racks.

Order-specific due dates add a time-sensitive requirement to these decisions. Due-date-aware picking has been studied in parts-to-picker systems [12], and specified due dates have also been incorporated into integrated RMFS decisions [13]. However, finite rack retention and order-specific due dates have received less attention within the same order–rack sequencing setting.

This paper studies this problem in a single-station RMFS with a finite rack buffer area. Given order requirements, rack inventory, picking station capacity, and buffer capacity, we jointly determine the order entry sequence, rack service sequence, and buffer retention decisions. A mixed-integer programming model minimizes rack movements and weighted order tardiness. For larger instances, we develop a deadline-aware interactive heuristic with neighborhood search (DA-IHNS), in which normalized order urgency and rack coverage guide constructive decisions and order- and rack-level neighborhoods improve the resulting solution.

The main contributions of this paper are summarized as follows.

(1) We formulate an order and rack sequencing model that jointly considers order-specific due dates and finite rack-buffer retention. By linking order entry, rack service, and buffer retention decisions, the model captures the trade-off between rack reuse and weighted order tardiness under limited buffer capacity.
(2) We develop DA-IHNS to coordinate rack reuse and order urgency within the solution process. The constructive rules combine normalized urgency, rack coverage, and buffer retention value, while the neighborhood search operates on both the order sequence and selected rack service decisions.
(3) We evaluate the model and algorithm through exact validation, paired computational comparisons, and sensitivity analyses. The experiments assess solution quality, the contributions of the main algorithmic components, and the trade-off between rack reuse and order timeliness across the tested settings.

## 2. Literature review

### 2.1. RMFS operational optimization and integrated decision-making

Robotic mobile fulfillment systems represent a flexible parts-to-picker configuration in which movable racks are transported to stationary picking stations. Reviews by Azadeh et al. [5] and da Costa Barros and Nascimento [6], together with performance-oriented RMFS research [14], summarize the operational characteristics and planning problems of robotic warehouses, including order assignment, rack handling, robot scheduling, storage decisions, and workstation operations. Recent warehousing research further emphasizes the growing importance of coordinating automated resources and interdependent planning decisions [15].

Such coordination is particularly important when multiple operational decisions share limited workstation and material-handling resources. Van Gils et al. [16] show that batching, assignment, sequencing, routing, and resource allocation are closely related in order-picking planning. In parts-to-picker systems, orders and inventory carriers must be synchronized at shared workstations [10]. RMFS studies extend this coordination to orders, racks, robots, and workstations [17–19]. Within this broader setting, coordination between order processing and rack service at the picking station constitutes a central operational problem.

### 2.2. Order and rack sequencing, rack reuse, and buffer-related decisions

Order and rack sequencing determines how orders processed at a workstation are coordinated with the racks required to fulfill them. Boysen et al. [8] formalize this synchronization at a rack-moving picking station and show the interdependence between order processing and rack visits. Yang et al. [9] formulate the joint optimization of order sequencing and rack scheduling in RMFSs, while Justkowiak and Pesch [20] strengthen the mixed-integer formulation for the single-station order- and rack-sequencing problem. Extensions to multiple-workstation settings incorporate additional order and rack coordination decisions [21,22], while more recent work considers further assignment and sequencing decisions in KIVA picking systems [23].

Rack allocation and storage decisions also influence subsequent rack handling. Valle and Beasley [24] jointly consider order and rack allocation together with rack sequencing, while Roy et al. [25], Weidinger et al. [26], and Shi et al. [27] examine rack storage, retrieval, and assignment decisions.

Temporary rack retention provides a more direct mechanism for rack reuse. Han et al. [11] study an order and rack sequencing problem with a finite rack buffer area and jointly determine the order picking sequence, rack retrieval sequence, and rack buffering strategy to reduce rack retrievals. Related temporary-storage mechanisms appear in other automated picking configurations. Zheng et al. [28] consider order sequencing and temporary rack shelving in a separated bin-picking system, while Wei et al. [29] coordinate order processing, bin assignment, and buffer-position assignment in a cooperative-robot picking system. Existing buffer-related studies therefore primarily emphasize rack reuse and material-movement reduction.

### 2.3. Due-date-aware and time-sensitive order fulfillment

Due dates connect warehouse picking decisions with time-sensitive fulfillment requirements. In conventional order-picking systems, Scholz et al. [30] integrate due dates with order batching, batch assignment and sequencing, and picker routing. Mohring et al. [31] examine cutoff-based shipment promises under warehouse capacity constraints, while Jiang et al. [32] jointly consider order assignment, sequencing, picking, and delivery under due dates.

Due-date requirements have also been incorporated into parts-to-picker and robotic warehouse operations. Zhao et al. [12] minimize total order tardiness through coordinated order assignment, sequencing, and rack-visit decisions in a parts-to-picker system. Hashemi and Ranjbar [13] consider specified due dates together with order sequencing, rack allocation, rack scheduling, and mobile robot routing in an RMFS. Zhang et al. [33] use dynamic order priorities to support time-sensitive order processing in robotic warehouse systems. These studies integrate order timeliness with assignment, sequencing, rack scheduling, and routing decisions, while finite rack retention is not their central operational mechanism.

### 2.4. Solution approaches for coupled order–rack decisions and research positioning

Order- and rack-sequencing problems have been addressed through both exact formulations and problem-specific solution procedures. Justkowiak and Pesch [20] develop a strengthened mixed-integer formulation for the single-station problem, while related RMFS scheduling decisions have also been formulated through integrated optimization models [34]. For larger instances, Justkowiak and Pesch [35] propose a column-generation-driven heuristic that constructs order batches while reducing the rack assignments required to serve them. Justkowiak et al. [36] coordinate order scheduling intervals and rack visit sequences through dynamic programming. Recent work further considers integrated order–rack sequencing under multi-visit mobile-robot operations [37].

Neighborhood-based and problem-specific search methods provide another approach to robotic fulfillment decisions. Gharehgozli and Zaerpour [38] address pod retrieval scheduling in RMFSs, while Zhang et al. [39] apply variable neighborhood search to an integrated order and robot scheduling problem. Shi et al. [27] embed variable neighborhood search in a bi-level rack sequencing and storage assignment framework. Rule-based procedures are also used for rapid operational decisions; Merschformann et al. [40] evaluate decision rules for several RMFS subproblems under system-specific interactions.

Existing rack-buffer studies primarily address rack reuse and movement reduction, whereas due-date-aware studies mainly incorporate order timeliness into assignment, sequencing, rack scheduling, routing, or delivery decisions. The joint consideration of finite rack retention and order-specific due dates within a single-station order–rack sequencing problem remains less developed. This study addresses this setting by coordinating order urgency, rack service, and rack reuse within the same optimization framework.

## 3. Problem description and model formulation

### 3.1. Problem description

This paper studies a single-station order and rack sequencing problem with a rack buffer area. The system processes a set of orders within a planning horizon. Each order contains demands for multiple products and has a given due date. Products are stored on movable racks. Each rack can store multiple products, and the inventory quantity of each product on each rack is known. Racks selected for service are transported by mobile robots to the picking station, where products are picked for the orders currently assigned to the station.

The capacity of the picking station is limited, and only a certain number of order totes can be placed at the station at the same time. Once an order is completed, it leaves the picking station, and a new order is added according to the order entry sequence. A rack buffer area with limited capacity is located near the picking station. After a retrieved rack completes its current picking task, if it may still be needed by subsequent orders and the buffer area has available space, it can be temporarily retained in the buffer area; otherwise, it returns to the storage area. This configuration is consistent with related studies on single-station order–rack coordination and rack buffering in parts-to-picker and RMFS settings [8,9,11].

Throughout this paper, rack movements are measured as rack retrievals from storage. Each retrieval from storage counts as one rack movement, whereas reuse of a rack retained in the buffer does not incur an additional retrieval.

The planning horizon is represented by discrete time slots, each corresponding to one rack service opportunity at the picking station. Order completion times and due dates are measured in the same time-slot units.

If an order is completed after its due date, order tardiness occurs. The system therefore coordinates rack reuse and order timeliness. The main decisions are the order entry sequence, rack service sequence, and buffer retention decisions. The objective is to minimize the number of rack retrievals from storage and weighted order tardiness, subject to order requirements, rack inventory, picking station capacity, and buffer capacity.

### 3.2. Model assumptions and notation

To facilitate model formulation, the following assumptions are made.

(1) All order information is known at the beginning of the planning horizon, including product types, demand quantities, and order due dates.
(2) All demand of an order is fulfilled at the considered picking station, possibly over multiple rack-service slots.
(3) In each time slot, the picking station can serve at most one rack, and the picker can retrieve products from that rack for multiple orders at the picking station.
(4) The rack buffer area has limited capacity, and at most a given number of racks can be stored in the buffer area at the same time.
(5) The model focuses on order sequencing, rack service sequencing, and buffer retention decisions. Rack-specific transportation times and robot path congestion are abstracted by the rack-service time-slot representation, and replenishment operations are not considered. Consequently, the reported completion times represent rack-service-slot completion times rather than physical travel-time-based completion times. The results are therefore interpreted under a common rack-service-time abstraction.

The main sets, parameters, and decision variables used in this paper are shown in Table 1.

**Table 1:** Notation used in the model.

Symbol	Description
**Sets and indices**	
O={1,2,...,N}	Set of orders, where n∈O denotes an order index
ℛ={1,2,...,K}	Set of racks, where k∈ℛ denotes a rack index
𝒮={1,2,...,S}	Set of product types, where s∈𝒮 denotes a product index
$𝒫=\{1,2,...,P_{\max}\}$	Set of rack-service time slots, where p∈𝒫 denotes a time slot and $P_{\max}=\sum_{n\in O}\sum_{s\in 𝒮}d_{ns}$
ℱ	Set of feasible order–rack–product triples (*n*,*k*,*s*) for which $d_{ns}>0$ and $q_{ks}>0$
**Parameters**	
*C*	Capacity of the picking station, i.e., the maximum number of orders that can be processed simultaneously at the picking station in each time slot
*H*	Capacity of the rack buffer area, i.e., the maximum number of racks that can be stored in the buffer area at the same time
$d_{ns}$	Demand quantity of order *n* for product *s*
$q_{ks}$	Inventory quantity of product *s* on rack *k*
$D_{n}$	Due date of order *n*
$w_{n}$	Tardiness weight of order *n*, where a larger value indicates a higher penalty for order tardiness
λ	Tardiness penalty coefficient, used to balance rack retrievals and weighted order tardiness
**Decision variables**	
$x_{np}$	Binary variable equal to 1 if order *n* occupies a position at the picking station in time slot *p*, and 0 otherwise
$v_{np}$	Binary variable equal to 1 if order *n* is processed in time slot *p*, and 0 otherwise
$y_{kp}$	Binary variable equal to 1 if rack *k* is used for picking in time slot *p*, and 0 otherwise
$u_{kp}$	Binary variable equal to 1 if rack *k* is retrieved from the storage area in time slot *p*, and 0 otherwise
$b_{kp}$	Binary variable equal to 1 if rack *k* is retained in the buffer area after time slot *p*, and 0 otherwise
$e_{np}$	Binary variable equal to 1 if order *n* enters the picking station in time slot *p*, and 0 otherwise
$h_{np}$	Binary variable equal to 1 if order *n* is completed in time slot *p*, and 0 otherwise
$z_{nkps}$	Non-negative integer variable representing the quantity of product *s* picked from rack *k* for order *n* in time slot *p*, defined for (n,k,s)∈ℱ
$C_{n}$	Continuous variable representing the rack-service-slot completion time of order *n*
$T_{n}$	Continuous variable representing the tardiness of order *n*

### 3.3. Mixed-integer programming model

Based on the above problem description and related order–rack formulations [9,11,20], this paper formulates a mixed-integer programming model for the order and rack sequencing problem with a rack buffer area under order due dates. The objective minimizes rack retrievals from storage and weighted order tardiness. Each productive rack-service slot contains at least one unit of picking activity, while the total quantity picked over the planning horizon equals $\sum_{n\in O}\sum_{s\in 𝒮}d_{ns}$. Since nonproductive idle slots can be removed without worsening the objective,


$$\begin{array}{c} P_{\max}=\sum_{n\in O}\sum_{s\in 𝒮}d_{ns} \end{array}$$ (1)

provides a valid upper bound on the number of productive rack-service slots. The model is given as follows:


$$\begin{array}{c} \min Z=\sum_{k\in ℛ}\sum_{p\in 𝒫}u_{kp}+\lambda \sum_{n\in O}w_{n}T_{n}. \end{array}$$ (2)

In Eq (2), the first term counts storage retrievals of racks, and the second term represents total weighted order tardiness. The tardiness penalty coefficient λ controls their relative importance.

**(1) Picking station capacity constraints.** In any time slot, the number of orders processed simultaneously at the picking station cannot exceed the station capacity:


$$\begin{array}{c} \sum_{n\in O}x_{np}\leq C,\;\forall p\in 𝒫. \end{array}$$ (3)

**(2) Order entry, completion, and continuity constraints.** Each order enters and completes processing exactly once:


$$\begin{array}{c} \sum_{p\in 𝒫}e_{np}=1,\;\forall n\in O, \end{array}$$ (4)

$$\begin{array}{c} \sum_{p\in 𝒫}h_{np}=1,\;\forall n\in O. \end{array}$$ (5)

The occupancy state begins when the order enters the station and ends immediately after its completion slot:


$$\begin{array}{c} x_{n1}=e_{n1},\;\forall n\in O, \end{array}$$ (6)

$$\begin{array}{c} x_{np}=x_{n,p-1}+e_{np}-h_{n,p-1},\;\forall n\in O,\;p=2,...,P_{\max}, \end{array}$$ (7)

$$\begin{array}{c} h_{nP_{\max}}=x_{nP_{\max}},\;\forall n\in O. \end{array}$$ (8)

A completion indicator can be activated only in a slot in which the corresponding order is processed, while processing requires the order to be present at the station:


$$\begin{array}{c} h_{np}\leq v_{np},\;\forall n\in O,\;p\in 𝒫, \end{array}$$ (9)

$$\begin{array}{c} v_{np}\leq x_{np},\;\forall n\in O,\;p\in 𝒫. \end{array}$$ (10)

Eqs (4)–(10) provide a compact time-indexed representation of consecutive order occupancy and prevent an order from leaving and subsequently re-entering the picking station.

**(3) Order demand satisfaction constraints.** The demand for each product in each order must be fully satisfied:


$$\begin{array}{c} \sum_{\begin{array}{c} k\in ℛ:(n,k,s)\in ℱ \end{array}}\sum_{p\in 𝒫}z_{nkps}=d_{ns},\;\forall n\in O,\;s\in 𝒮:d_{ns}>0. \end{array}$$ (11)

**(4) Rack inventory constraints.** The quantity of products picked from any rack cannot exceed the inventory quantity of the corresponding products on that rack:


$$\begin{array}{c} \sum_{\begin{array}{c} n\in O:(n,k,s)\in ℱ \end{array}}\sum_{p\in 𝒫}z_{nkps}\leq q_{ks},\;\forall k\in ℛ,\;s\in 𝒮:q_{ks}>0. \end{array}$$ (12)

**(5) Linking constraints among orders, racks, and picked quantities.** Products can be picked only when the corresponding order is at the picking station and the corresponding rack is being served. Let ${\bar{q}}_{nks}=\min\{d_{ns},q_{ks}\}$ for (n,k,s)∈ℱ. Then


$$\begin{array}{c} z_{nkps}\leq {\bar{q}}_{nks}x_{np},\;\forall (n,k,s)\in ℱ,\;p\in 𝒫, \end{array}$$ (13)

$$\begin{array}{c} z_{nkps}\leq {\bar{q}}_{nks}y_{kp},\;\forall (n,k,s)\in ℱ,\;p\in 𝒫, \end{array}$$ (14)

$$\begin{array}{c} z_{nkps}\leq {\bar{q}}_{nks}v_{np},\;\forall (n,k,s)\in ℱ,\;p\in 𝒫. \end{array}$$ (15)

Conversely, an order-processing indicator or rack-service indicator can be active only if positive picking occurs in the corresponding time slot:


$$\begin{array}{c} v_{np}\leq \sum_{\begin{array}{c} k\in ℛ,\;s\in 𝒮:\\ (n,k,s)\in ℱ \end{array}}z_{nkps},\;\forall n\in O,\;p\in 𝒫, \end{array}$$ (16)

$$\begin{array}{c} y_{kp}\leq \sum_{\begin{array}{c} n\in O,\;s\in 𝒮:\\ (n,k,s)\in ℱ \end{array}}z_{nkps},\;\forall k\in ℛ,\;p\in 𝒫. \end{array}$$ (17)

Eqs (13)–(15) permit positive picking only when the order is present and processed and the corresponding rack is being served. Eqs (16) and (17) exclude nonproductive order-processing and rack-service states.

**(6) Single-rack service constraints in each time slot.** In any time slot, the picking station can serve at most one rack:


$$\begin{array}{c} \sum_{k\in ℛ}y_{kp}\leq 1,\;\forall p\in 𝒫. \end{array}$$ (18)

**(7) Rack retrieval and buffer source constraints.** If a rack is used in time slot *p*, then the rack is either retrieved from the storage area or comes from the buffer area retained after the previous time slot. In the first time slot, any rack used can only come from the storage area:


$$\begin{array}{c} y_{k1}=u_{k1},\;\forall k\in ℛ. \end{array}$$ (19)

For the subsequent time slots, we have:


$$\begin{array}{c} y_{kp}\leq u_{kp}+b_{k,p-1},\;\forall k\in ℛ,\;p=2,...,P_{\max}, \end{array}$$ (20)

$$\begin{array}{c} u_{kp}\leq y_{kp},\;\forall k\in ℛ,\;p\in 𝒫, \end{array}$$ (21)

$$\begin{array}{c} u_{kp}\leq 1-b_{k,p-1},\;\forall k\in ℛ,\;p=2,...,P_{\max}. \end{array}$$ (22)

Eq (22) prevents a rack already available in the buffer from being counted as a new storage retrieval.

**(8) Rack buffer capacity constraints.** After each time slot, the number of racks retained in the buffer area cannot exceed the buffer capacity:


$$\begin{array}{c} \sum_{k\in ℛ}b_{kp}\leq H,\;\forall p\in 𝒫. \end{array}$$ (23)

In addition, only a rack that is currently used or has already been retained in the buffer area after the previous time slot can continue to be retained in the buffer area:


$$\begin{array}{c} b_{k1}\leq y_{k1},\;\forall k\in ℛ, \end{array}$$ (24)

$$\begin{array}{c} b_{kp}\leq y_{kp}+b_{k,p-1},\;\forall k\in ℛ,\;p=2,...,P_{\max}. \end{array}$$ (25)

To avoid ineffective buffering, a rack will not be retained in the buffer area if it will not be used again in subsequent time slots:


$$\begin{array}{c} b_{kp}\leq \sum_{\tau =p+1}^{P_{\max}}y_{k\tau },\;\forall k\in ℛ,\;p=1,...,P_{\max}-1, \end{array}$$ (26)

$$\begin{array}{c} b_{kP_{\max}}=0,\;\forall k\in ℛ. \end{array}$$ (27)

**(9) Order completion time and tardiness constraints.** The unique completion indicator determines the rack-service-slot completion time of each order:


$$\begin{array}{c} C_{n}=\sum_{p\in 𝒫}p\;h_{np},\;\forall n\in O. \end{array}$$ (28)

The tardiness of an order is the positive part by which its completion time exceeds its due date:


$$\begin{array}{c} T_{n}\geq C_{n}-D_{n},\;\forall n\in O, \end{array}$$ (29)

$$\begin{array}{c} T_{n}\geq 0,\;\forall n\in O. \end{array}$$ (30)

**(10) Variable domains.**


$$\begin{array}{c} x_{np},v_{np},e_{np},h_{np}\in \{0,1\},\;\forall n\in O,\;p\in 𝒫, \end{array}$$ (31)

$$\begin{array}{c} y_{kp},u_{kp},b_{kp}\in \{0,1\},\;\forall k\in ℛ,\;p\in 𝒫, \end{array}$$ (32)

$$\begin{array}{c} z_{nkps}\in {\mathbb{Z}}_{+},\;\forall (n,k,s)\in ℱ,\;p\in 𝒫, \end{array}$$ (33)

$$\begin{array}{c} C_{n}\geq 0,\;T_{n}\geq 0,\;\forall n\in O. \end{array}$$ (34)

For computational implementation, picked-quantity variables are instantiated only for triples in ℱ. This sparsification removes variables that are necessarily zero and does not alter the feasible solution set.

The model jointly represents rack reuse efficiency and order timeliness through the two components of the objective function. When λ=0, the objective reduces to minimizing rack retrievals from storage; when λ>0, weighted order tardiness is also penalized.

## 4. DA-IHNS algorithm

### 4.1. Algorithmic framework

The problem involves three interdependent decisions: the order entry sequence, rack service sequence, and buffer retention decisions. To obtain feasible solutions efficiently, we develop a deadline-aware interactive heuristic with neighborhood search, denoted as DA-IHNS. The algorithm follows a construction–decoding–improvement framework. The construction stage simulates order admission, rack service, picking, and buffer updates to generate an order-admission chronology. This chronology defines the initial order sequence; the rack service and buffer states produced during construction are not treated as the final solution. The resulting order sequence is subsequently evaluated by the decoder and improved through order- and rack-level neighborhood search.

Let *UO* denote the set of orders that have not yet entered the picking station, *PO* denote the set of orders currently at the picking station, *B* denote the set of racks currently stored in the buffer area, and *p* denote the current time slot. Let $d_{ns}^{\prime }$ denote the remaining demand of order *n* for product *s*, and let $q_{ks}^{\prime }$ denote the remaining inventory of product *s* on rack *k*. During the algorithm, $d_{ns}^{\prime }$ and $q_{ks}^{\prime }$ are dynamically updated as the picking process proceeds.

### 4.2. Normalized scoring components

DA-IHNS combines order urgency, rack coverage, order completion contribution, and rack retrieval status in its decision rules. All components entering the weighted scoring functions are dimensionless and bounded within [0,1].

Given order *n* and rack *k*, the coverage of rack *k* for order *n* is defined as:


$$\begin{array}{c} MR(n,k)=\frac{\sum_{s\in 𝒮}\min\{q_{ks}^{\prime },d_{ns}^{\prime }\}}{\sum_{s\in 𝒮}d_{ns}^{\prime }},\;\sum_{s\in 𝒮}d_{ns}^{\prime }>0. \end{array}$$ (35)

*MR*(*n*,*k*) measures the proportion of the remaining demand of order *n* that can be supplied by rack *k*.

For a set of available racks ${ℛ}_{c}$, its overall coverage for order *n* is defined as:


$$\begin{array}{c} MR(n,{ℛ}_{c})=\frac{\sum_{s\in 𝒮}\min\left\{\sum_{k\in {ℛ}_{c}}q_{ks}^{\prime },d_{ns}^{\prime }\right\}}{\sum_{s\in 𝒮}d_{ns}^{\prime }},\;{ℛ}_{c}\neq \emptyset . \end{array}$$ (36)

This measure represents the joint coverage of the racks currently available for reuse.

The raw urgency of order *n* in time slot *p* is defined as


$$\begin{array}{c} Urg(n,p)=\frac{w_{n}}{1+\max\{D_{n}-p,0\}}. \end{array}$$ (37)

A larger value reflects a closer due date, a larger tardiness weight, or both. For each decision stage, urgency is normalized over the relevant candidate order set ${𝒜}_{p}$:


$$\begin{array}{c} \tilde{Urg}(n,p)=\left\{ \begin{array}{cc} \frac{Urg(n,p)}{\max_{j\in {𝒜}_{p}}Urg(j,p)}, & \max_{j\in {𝒜}_{p}}Urg(j,p)>0,\\ 0, & \text{otherwise}. \end{array}\right. \end{array}$$ (38)

The candidate set ${𝒜}_{p}$ is *UO* for order admission and *PO* for rack service and within-rack picking decisions.

For rack service, average coverage over the active orders is


$$\begin{array}{c} \overset{―}{MR}(k,p)=\frac{1}{\left|PO\right|}\sum_{n\in PO}MR(n,k),\;|PO|>0, \end{array}$$ (39)

and urgency-weighted coverage is


$$\begin{array}{c} UM(k,p)=\frac{1}{\left|PO\right|}\sum_{n\in PO}\tilde{Urg}(n,p)MR(n,k),\;|PO|>0. \end{array}$$ (40)

The completion contribution of rack *k* is measured by the proportion of active orders whose entire remaining demand can be satisfied by that rack:


$$\begin{array}{c} \tilde{Comp}(k,p)=\frac{1}{\left|PO\right|}\sum_{n\in PO}𝕀\left(\sum_{s\in 𝒮}\max\{d_{ns}^{\prime }-q_{ks}^{\prime },0\}=0\right),\;|PO|>0, \end{array}$$ (41)

where 𝕀(·) is an indicator function.

Rack retrieval status is represented by


$$\begin{array}{c} Out(k,p)=\left\{ \begin{array}{cc} 0, & k\in B,\\ 1, & k\notin B, \end{array}\right. \end{array}$$ (42)

where *Out*(*k*,*p*)=1 indicates that rack *k* requires retrieval from storage. The scoring components are therefore expressed on a common [0,1] scale. The parameters α, θ, δ, η, and ξ determine their relative contributions to the corresponding decision rules.

### 4.3. Deadline-aware order sequencing

Whenever the picking station has available capacity, candidate orders in *UO* are evaluated using normalized urgency and rack coverage. The coverage component is defined as


$$\begin{array}{c} Cov(n,p)=\left\{ \begin{array}{cc} MR(n,B), & B\neq \emptyset ,\\ \max_{k\in ℛ}MR(n,k), & B=\emptyset . \end{array}\right. \end{array}$$ (43)

The unified order-admission score is


$$\begin{array}{c} OS(n,p)=\alpha \tilde{Urg}(n,p)+(1-\alpha )Cov(n,p),\;n\in UO, \end{array}$$ (44)

where α∈[0,1] controls the relative emphasis on order urgency and rack coverage. The order with the largest score is admitted:


$$\begin{array}{c} n^{*}=\underset{n\in UO}{arg\;max}OS(n,p). \end{array}$$ (45)

The first admitted order is the special case with B=∅. Orders are subsequently admitted using the same rule until the picking station reaches capacity or no unassigned order remains.

### 4.4. Rack service and buffer retention

When unfinished orders are present at the picking station, only racks that can satisfy part of the remaining demand of at least one active order are eligible for service. The eligible buffered racks are


$$\begin{array}{c} B^{s}(p)=\left\{k\in B|\sum_{n\in PO}MR(n,k)>0\right\}, \end{array}$$ (46)

and the eligible racks outside the current buffer are


$$\begin{array}{c} {ℛ}^{s}(p)=\left\{k\in ℛ⧵B|\sum_{n\in PO}MR(n,k)>0\right\}. \end{array}$$ (47)

The candidate rack set is therefore


$$\begin{array}{c} {ℛ}^{can}=B^{s}(p)\cup {ℛ}^{s}(p). \end{array}$$ (48)

Using the normalized components defined in Section 4.2, the rack service score is


$$\begin{array}{c} SS(k,p)=\overset{―}{MR}(k,p)+\theta UM(k,p)+\delta \tilde{Comp}(k,p)-\eta Out(k,p),\;k\in {ℛ}^{can}. \end{array}$$ (49)

The terms represent average rack coverage, urgency-weighted coverage, completion contribution, and storage retrieval status, respectively. The rack selected for service is


$$\begin{array}{c} k^{*}=\underset{k\in {ℛ}^{can}}{arg\;max}SS(k,p). \end{array}$$ (50)

After rack $k^{*}$ is selected, orders in *PO* are ranked according to normalized urgency and rack coverage. With ${𝒜}_{p}=PO$, the priority score is


$$\begin{array}{c} PS(n,p|k^{*})=\frac{\tilde{Urg}(n,p)+\xi MR(n,k^{*})}{1+\xi },\;n\in PO. \end{array}$$ (51)

The factor 1+ξ scales the score without changing the ranking of candidate orders. The picker retrieves products for orders in descending order of $PS(n,p|k^{*})$. For order *n* and product *s*, the quantity that can be picked in the current time slot is:


$$\begin{array}{c} g_{ns}=\min\{d_{ns}^{\prime },q_{k^{*}s}^{\prime }\}. \end{array}$$ (52)

After picking, the remaining demand and rack inventory are updated as:


$$\begin{array}{c} d_{ns}^{\prime }\leftarrow d_{ns}^{\prime }-g_{ns},\;q_{k^{*}s}^{\prime }\leftarrow q_{k^{*}s}^{\prime }-g_{ns}. \end{array}$$ (53)

If all remaining demands of order *n* become zero, then the picking process of this order is completed, its completion time is recorded, and the order is removed from the picking station.

After rack service, the buffer is updated according to the reuse value of each candidate rack. Let ${𝒜}_{p}=UO\cup PO$ denote the unfinished orders. The retention value of rack *k* is


$$\begin{array}{c} Keep(k,p)=\frac{1}{\left|{𝒜}_{p}\right|}\sum_{n\in {𝒜}_{p}}\left[MR(n,k)+\theta \tilde{Urg}(n,p)MR(n,k)\right],\;|{𝒜}_{p}|>0. \end{array}$$ (54)

The candidate buffer set is $B^{can}=B\cup \{k^{*}\}$. Racks with positive retention values are ranked by *Keep*(*k*,*p*), and at most the top *H* racks are retained. The remaining racks return to storage. Thus, *H* specifies an upper bound on buffer occupancy rather than a required occupancy level.

### 4.5. Order- and rack-level neighborhood search

The order-level search operates on the order entry sequence using three neighborhood operators: swap, insertion, and tardy-order forward movement. The operators are applied cyclically, and the positions involved in each move are selected using the reproducible random number generator described in Section 4.6.

For the swap operator, two distinct positions are selected randomly and the corresponding orders are exchanged. For the insertion operator, one order is removed from a randomly selected position and inserted at another randomly selected position. The tardy-order forward operator identifies orders with positive tardiness in the incumbent solution, randomly selects one of them, and moves it toward the beginning of the sequence. If the selected order is at position *i* > 1, its maximum forward displacement is


$$\begin{array}{c} \min\left\{i-1,\;\max\left\{2,\left\lfloor N/5\right\rfloor \right\}\right\}. \end{array}$$ (55)

If the selected order is already in the first position, a swap move is generated. If no tardy order exists, an insertion move is used instead. Each candidate order sequence is evaluated by the decoding procedure described in Sections 4.4 and 4.6.

Rack-level intensification perturbs selected rack service decisions while keeping the order sequence fixed. The standard decoder ranks feasible racks according to Eq (49). Slots near the completion times of tardy orders are examined first, supplemented by approximately evenly spaced slots over the rack service sequence. At each selected slot, the second- or third-ranked feasible rack is tested in place of the highest-ranked rack, and the resulting plan is fully re-decoded. The search considers the top three feasible rack candidates and evaluates at most 12 rack-override trials in each invocation.

Rack-level trials use sequential strict-improvement acceptance. An improving feasible plan immediately becomes the incumbent rack plan, and the remaining trials continue from that plan until the trial limit is reached. At both neighborhood levels, a candidate is accepted only if


$$\begin{array}{c} Z^{new}<Z^{inc}. \end{array}$$ (56)

The neighborhood search therefore preserves the incumbent objective value when no strict improvement is found.

### 4.6. DA-IHNS procedure and termination

Algorithm 1 summarizes the construction, sequence evaluation, and neighborhood-search procedure of DA-IHNS (Table 2).

**Table 2:** Algorithm 1. DA-IHNS.

Step	Procedure
1	Input the order demand matrix, rack inventory matrix, order due dates, picking station capacity *C*, rack buffer capacity *H*, and algorithm parameters. Initialize the reproducible random number generator.
2	Set *UO* = *O*, PO=∅, B=∅, and *p* = 1. Initialize the remaining order demands and rack inventories for the construction simulation.
3	Admit orders successively according to Eqs (43)–(45) until the picking station reaches capacity or UO=∅.
4	Construct ${ℛ}^{can}$, select rack $k^{*}$ according to Eqs (49) and (50), and perform picking according to Eq (51).
5	Update the remaining demands, rack inventories, completed orders, and buffer state. Set p←p+1 and repeat Steps 3–5 until all orders are completed.
6	Use the resulting admission chronology as the initial order sequence. Evaluate this sequence with the decoder. When *H* > 0, also evaluate the same sequence with *H* = 0 and retain the feasible solution with the lower objective value.
7	Apply rack-level intensification to the initial incumbent using the top three feasible rack candidates and at most 12 rack-override trials.
8	Generate an order-level neighbor using swap, insertion, and tardy-order forward movement cyclically, and evaluate the candidate sequence using the procedure in Step 6.
9	If the candidate strictly improves the incumbent, apply rack-level intensification to the candidate before accepting it. Otherwise, retain the incumbent and increase the consecutive non-improvement counter.
10	Every 10 order-neighborhood iterations, apply rack-level intensification to the current incumbent. Reset the consecutive non-improvement counter if an improvement is obtained.
11	Repeat Steps 8–10 until the iteration limit or the consecutive non-improvement limit is reached.
12	Output the order entry sequence, rack service sequence, buffer retention plan, order completion times, rack retrieval count, weighted order tardiness, and comprehensive objective value.

During order-sequence evaluation, the buffered decode and, when *H* > 0, the corresponding *H* = 0 decode are compared for the same order sequence, and the feasible solution with the lower comprehensive objective value is retained. This comparison treats buffer capacity as an available resource rather than mandatory buffer use.

The iteration and stagnation limits depend on the number of orders. The pairs $\left(I_{\max},I_{stall}\right)$ are (60,20) for N≤10, (80,25) for 10<N≤30, (100,30) for 30<N≤50, and (120,40) for *N* > 50. The order-level search terminates when either limit is reached.

Randomized order-neighborhood moves use the fixed algorithm seed 20260811 combined with the seed of each instance. Score ties are resolved by the smaller order or rack index. The same instance seeds and algorithm settings are used in paired comparisons.

By construction, every rack in ${ℛ}^{can}$ has positive coverage for at least one active order. Each selected rack therefore satisfies at least one unit of remaining demand. Since total demand is finite, the decoding process makes positive progress in every rack-service slot and terminates after a finite number of slots.

## 5. Computational experiments and results

### 5.1. Experimental design

#### 5.1.1. Instance generation and due-date construction.

The computational experiments use an instance bank comprising exact-validation instances, main-comparison instances, and a common set of sensitivity-analysis instances.

For the main and sensitivity instances, each order contains one to five product types with probabilities 0.35, 0.25, 0.18, 0.13, and 0.09, respectively. The demand quantity of each selected product is 1 or 2 with probabilities 0.82 and 0.18. Order tardiness weights take values 1, 2, and 3 with probabilities 0.60, 0.30, and 0.10. For each rack, the target number of stored product types is approximately 0.12*S* and is bounded between 3 and 30; the realized number varies by at most two product types around this target. Product types are sampled according to their demand frequencies, and the initial inventory quantity of each selected product is sampled from 1 to 4 units. If the aggregate inventory of a product is insufficient, additional units are assigned to randomly selected racks until product-level feasibility is restored. Exact-validation instances use at most three product types per order and a denser rack assortment to keep the mixed-integer programming model computationally tractable.

Order due dates are generated independently of any heuristic solution. Let *L* denote the total number of nonzero order–product demand lines. The basic due-date horizon is defined as


$$\begin{array}{c} \tau_{0}=\max\left\{3,\left\lceil \frac{L}{C}\right\rceil \right\}. \end{array}$$ (57)

For each order, the age $a_{n}$ is sampled uniformly from the integers between 0 and $\max\{1,\mathrm{round}(0.25\tau_{0})\}$. The promised-window factor $\phi_{n}$ takes values 0.70, 0.85, 1.00, 1.15, and 1.30 with probabilities 0.12, 0.23, 0.35, 0.20, and 0.10, respectively. A discrete disturbance $\varepsilon_{n}\in \{-1,0,1\}$ is generated with probabilities 0.20, 0.60, and 0.20. The due date is then generated as


$$\begin{array}{c} D_{n}=\max\left\{1,\mathrm{round}\left(\rho \tau_{0}\phi_{n}-a_{n}+\varepsilon_{n}\right)\right\}. \end{array}$$ (58)

A smaller due-date tightness coefficient ρ produces tighter due dates, and the baseline experiments use ρ=1.2. Due dates are generated before optimization and do not depend on the solution returned by any tested algorithm. All order information is therefore known when scheduling begins, consistent with the static setting in Section 3.

For reproducibility, all instances are generated from fixed seeds derived from the base seed 20260811. The same instances are reused in paired algorithm and parameter comparisons. In the due-date-tightness experiment, $a_{n}$, $\phi_{n}$, and $\varepsilon_{n}$ remain fixed for each base instance, and only ρ is varied in Eq (58).

The principal instance settings are summarized in Table 3. Entries separated by commas in the exact-validation row correspond by position.

**Table 3:** Experimental instance design.

Experiment	*N*	*K*	*S*	*C*	*H*	Instances
Exact validation	4, 6, 8, 10, 15, 20	8, 10, 12, 14, 18, 22	10, 15, 20, 20, 24, 28	2, 3, 4, 5, 7, 8	2, 2, 3, 3, 4, 5	10, 10, 10, 10, 5, 5
Main comparison	30, 50, 80	60, 100, 150	100, 150, 200	8	5	20 per size
Sensitivity base set	50	100	150	8	5	20

#### 5.1.2. Parameter settings, benchmark methods, and evaluation metrics.

The baseline computational settings of DA-IHNS are listed in Table 4. These values provide a common reference configuration for the paired experiments rather than an empirically calibrated optimum. Unless a parameter is explicitly varied, the same configuration is used across the corresponding experiments.

**Table 4:** Baseline computational settings.

Setting	Value	Role
Tardiness penalty coefficient λ	1.00	Relative penalty on weighted tardiness
Order urgency weight α	0.50	Order sequencing
Urgency–coverage weight θ	1.00	Rack service and buffer retention
Completion contribution weight δ	0.50	Rack service scoring
Rack retrieval penalty weight η	0.50	Rack service scoring
Rack coverage weight ξ	0.50	Picking priority
Rack-level candidate depth	Top 3	Rack-level neighborhood
Maximum rack-override trials	12	Rack-level neighborhood
Algorithm random seed	20260811	Reproducible neighborhood moves

The sensitivity of the heuristic scoring rules is examined on the same 10 paired sensitivity instances using one common baseline configuration and two one-factor variations for each of α, θ, δ, η, and ξ, giving 11 configurations in total. The low and high levels are α∈{0.25,0.75}, θ∈{0.50,1.50}, and δ,η,ξ∈{0.25,0.75}, with all unspecified parameters fixed at their baseline values. The results do not identify a uniformly dominant setting across the five scoring weights. The prespecified baseline configuration is retained throughout the main experiments, and the complete sensitivity results are reported in S2 Table.

The coefficient λ is a relative penalty coefficient in the composite objective and is not interpreted as an empirically calibrated monetary conversion factor between one rack movement and one unit of weighted tardiness. The value λ=1 is adopted as the baseline computational setting. Its influence is examined separately over λ∈{0.25,0.50,1.00,2.00,4.00} using the same 20 sensitivity instances.

The experiments were conducted on a computer equipped with an Intel(R) Core(TM) Ultra 9 275HX processor and Windows 11 (version 10.0.26100). The implementation used Python 3.10.9, NumPy 1.23.5, pandas 1.5.3, SciPy 1.10.0, DOcplex 2.23.221, and IBM ILOG CPLEX 22.1.0.0. CPLEX was run with one thread. Its time limit was 600 s for N≤10 and 300 s for *N* = 15 and 20.

IH and EDD-IH are used as the primary rule-based benchmarks. IH removes due-date information from the order-admission and rack-service scoring rules by setting α=θ=0 and does not use neighborhood search. EDD-IH fixes the order sequence in ascending order of due date, with the larger tardiness weight used as a tie breaker, and uses the same coverage-oriented rack-service configuration as IH. The common decoding and within-rack picking procedure is retained across both benchmarks. No-buffer DA-IHNS and DA-IHNS-OrderOnly are treated as ablation variants rather than primary benchmarks. The former sets *H* = 0, whereas the latter removes the rack-level neighborhood from the complete DA-IHNS.

The principal performance measure is the comprehensive objective value


$$\begin{array}{c} Z=R+\lambda WTT,\;WTT=\sum_{n\in O}w_{n}T_{n}, \end{array}$$ (59)

where *R* denotes rack movements, measured as rack retrievals from storage, and *WTT* denotes weighted order tardiness. CPU time is recorded in seconds. For CPLEX validation, the reported MIP gap is the solver-reported gap between the incumbent solution and its best bound. The relative deviation of DA-IHNS from the CPLEX objective is calculated as


$$\begin{array}{c} RD=\frac{Z_{DA}-Z_{CPLEX}}{Z_{CPLEX}}\times 100\%. \end{array}$$ (60)

When CPLEX proves optimality, *RD* measures the deviation from the optimum. Otherwise, it is interpreted only as the relative difference from the CPLEX incumbent. For paired heuristic comparisons, the objective reduction achieved by DA-IHNS is calculated as


$$\begin{array}{c} Improvement=\frac{Z_{base}-Z_{DA}}{Z_{base}}\times 100\%. \end{array}$$ (61)

Positive values indicate a lower objective value for DA-IHNS. Reported mean paired reductions are arithmetic means of the corresponding instance-level percentage reductions. Paired differences are evaluated using one-sided Wilcoxon signed-rank tests, with Holm adjustment across the reported paired tests. Where shown, error bars are normal-approximation 95% confidence intervals for the mean, with half-width $1.96s/\sqrt{n}$.

### 5.2. Exact-solution validation and computational efficiency

The exact-validation experiment evaluates DA-IHNS against the mixed-integer programming model over *N* = 4,6,8,10,15, and 20. Ten independent instances are used for each size up to *N* = 10, and five are used for *N* = 15 and 20. Table 5 reports scale-level averages. A CPLEX run is classified as proven optimal when CPLEX returns an optimal status and the reported relative MIP gap is zero within the numerical tolerance used in the implementation. Per-instance objective values, best bounds, solver status, MIP gaps, and CPU times are reported in S1 Table.

**Table 5:** Exact-solution validation and computational performance.

*N*	Runs	CPLEX status	$Z_{CPLEX}$	Bound	Gap (%)	$Z_{DA}$	*RD* (%)	CPLEX time (s)	DA time (s)
4	10	10/10 optimal	2.00	2.00	0.00	2.00	0.00	0.07	0.03
6	10	10/10 optimal	2.90	2.90	0.00	2.90	0.00	0.28	0.07
8	10	9/10 optimal; 1 gap	2.90	2.80	3.33	3.00	3.33	0.42	0.11
10	10	10/10 optimal	3.70	3.70	0.00	4.10	11.67	4.27	0.16
15	5	5/5 optimal	4.40	4.40	0.00	5.40	23.67	7.66	0.34
20	5	4/5 optimal; 1 time limit	5.00	4.80	4.00	6.20	24.00	152.29	0.64

Note: Values are averages within each problem size. Gap and *RD* values are averages of the corresponding instance-level percentages and are not recomputed from the displayed scale-level mean objective values or bounds. For runs with a positive CPLEX MIP gap, *RD* is a descriptive relative difference rather than an optimality gap.

DA-IHNS matches the CPLEX objective on all instances with *N* = 4 and 6. At *N* = 8, nine of the ten instances have matching objective values and the mean relative deviation is 3.33%. The deviation increases from *N* = 10 onward, reaching 11.67% at *N* = 10, 23.67% at *N* = 15, and 24.00% at *N* = 20. The exact-validation results therefore support the solution quality of DA-IHNS within the small-instance range indicated by these comparisons.

The computational contrast is most pronounced at *N* = 20. CPLEX requires an average of 152.29 s and reaches the 300 s time limit on one of five instances, whereas DA-IHNS requires an average of 0.64 s. This computational difference motivates the use of the heuristic in the larger paired experiments without extending the small-scale solution-quality claim to *N* = 15 or 20.

### 5.3. Comparative performance across problem sizes

The main comparison uses 60 fixed paired instances, with 20 instances each for *N* = 30, 50, and 80. DA-IHNS is compared with IH and EDD-IH on the same instances at each scale. The mean paired objective reduction relative to IH increases from 83.65% at *N* = 30 to 93.98% at *N* = 80. Relative to EDD-IH, the corresponding reductions are 79.86%, 85.78%, and 90.26%. DA-IHNS obtains a lower comprehensive objective value on all 60 paired instances against both rule-based benchmarks. The paired Wilcoxon signed-rank tests remain significant after Holm adjustment at each problem size (*p* < 0.001). Fig 1 summarizes the scale-level paired reductions. Scale-level values of *Z*, *R*, *WTT*, and CPU time for the three methods are reported in S4 Table.

**Fig 1:**
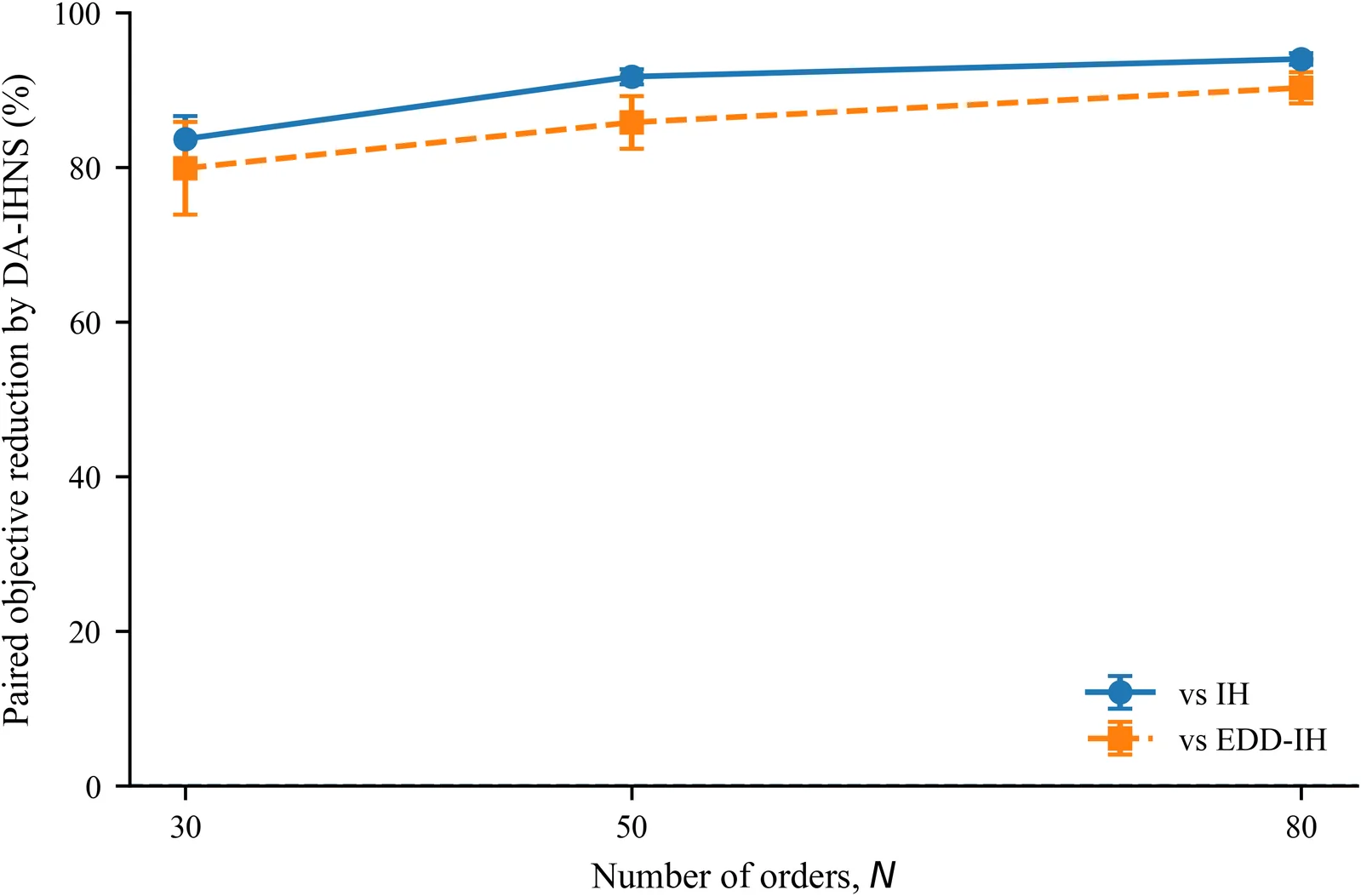
Paired objective reduction by DA-IHNS across problem sizes. Points show the mean paired reduction in the comprehensive objective value relative to IH and EDD-IH; error bars denote normal-approximation 95% confidence intervals for the mean.

Fig 2A shows that the paired reductions remain positive across the instance-level distributions.

**Fig 2:**
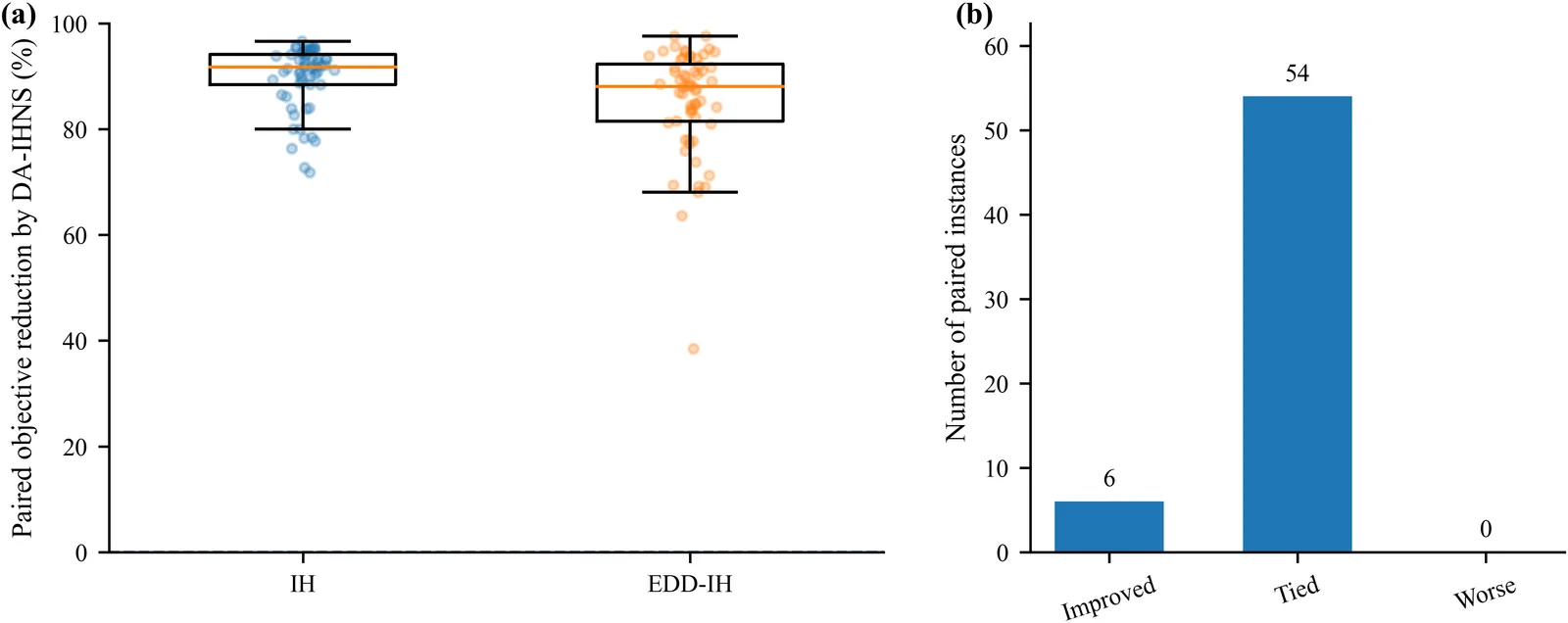
Instance-level paired comparisons and rack-level neighborhood ablation. (A) Distribution of paired objective reductions achieved by DA-IHNS relative to IH and EDD-IH over the 60 main-test instances. (B) Numbers of instances improved, tied, or worsened by the rack-level neighborhood relative to DA-IHNS-OrderOnly. Panel B is discussed in the ablation analysis.

### 5.4. Ablation analysis

To examine the contributions of rack buffering and rack-level neighborhood search, DA-IHNS is compared with two ablation variants on the same 60 main-test instances. No-buffer DA-IHNS retains the deadline-aware rules and neighborhood search but disables rack buffering. DA-IHNS-OrderOnly retains the complete decoding procedure but removes the rack-level neighborhood. Table 6 reports the paired results.

**Table 6:** Ablation analysis of DA-IHNS.

Variant	Mean *Z*	Mean *R*	Mean *WTT*	Paired reduction (%)	W/T/L
DA-IHNS	20.30	18.67	1.63	–	–
No-buffer DA-IHNS	20.82	20.03	0.79	1.66	32/6/22
DA-IHNS-OrderOnly	20.42	18.70	1.72	0.72	6/54/0

Note: Paired reduction is the mean percentage reduction in *Z* achieved by DA-IHNS relative to the corresponding ablation variant. W/T/L denotes the numbers of instances in which DA-IHNS obtains a lower, equal, or higher *Z*, respectively.

Removing the rack buffer increases the mean rack retrieval count from 18.67 to 20.03, while mean weighted tardiness decreases from 1.63 to 0.79. The complete DA-IHNS nevertheless obtains a lower mean comprehensive objective. The paired effect varies across instances, indicating that rack buffering primarily reduces repeated rack retrievals rather than uniformly improving both objective components.

As shown in Fig 2B, the rack-level neighborhood improves the comprehensive objective on six instances relative to DA-IHNS-OrderOnly and leaves 54 unchanged, with no deterioration. The rack-level stage therefore acts as a non-worsening intensification step that improves a limited subset of order-search incumbents.

### 5.5. Sensitivity to due-date tightness

The due-date tightness coefficient is varied over ρ∈{0.8,1.0,1.2,1.4,1.6} using the same 20 base instances at every level. For each instance, $a_{n}$, $\phi_{n}$, and $\varepsilon_{n}$ are held fixed, and only ρ is changed when the due dates are regenerated according to Eq (58). DA-IHNS, IH, and EDD-IH are evaluated on the same resulting instance at each level.

The mean paired objective reduction achieved by DA-IHNS remains positive throughout the tested range. Relative to IH, the mean reduction ranges from 81.57% to 91.02%. Relative to EDD-IH, it ranges from 59.67% to 88.72% and becomes smaller at the more relaxed due-date settings, as shown in Fig 3.

**Fig 3:**
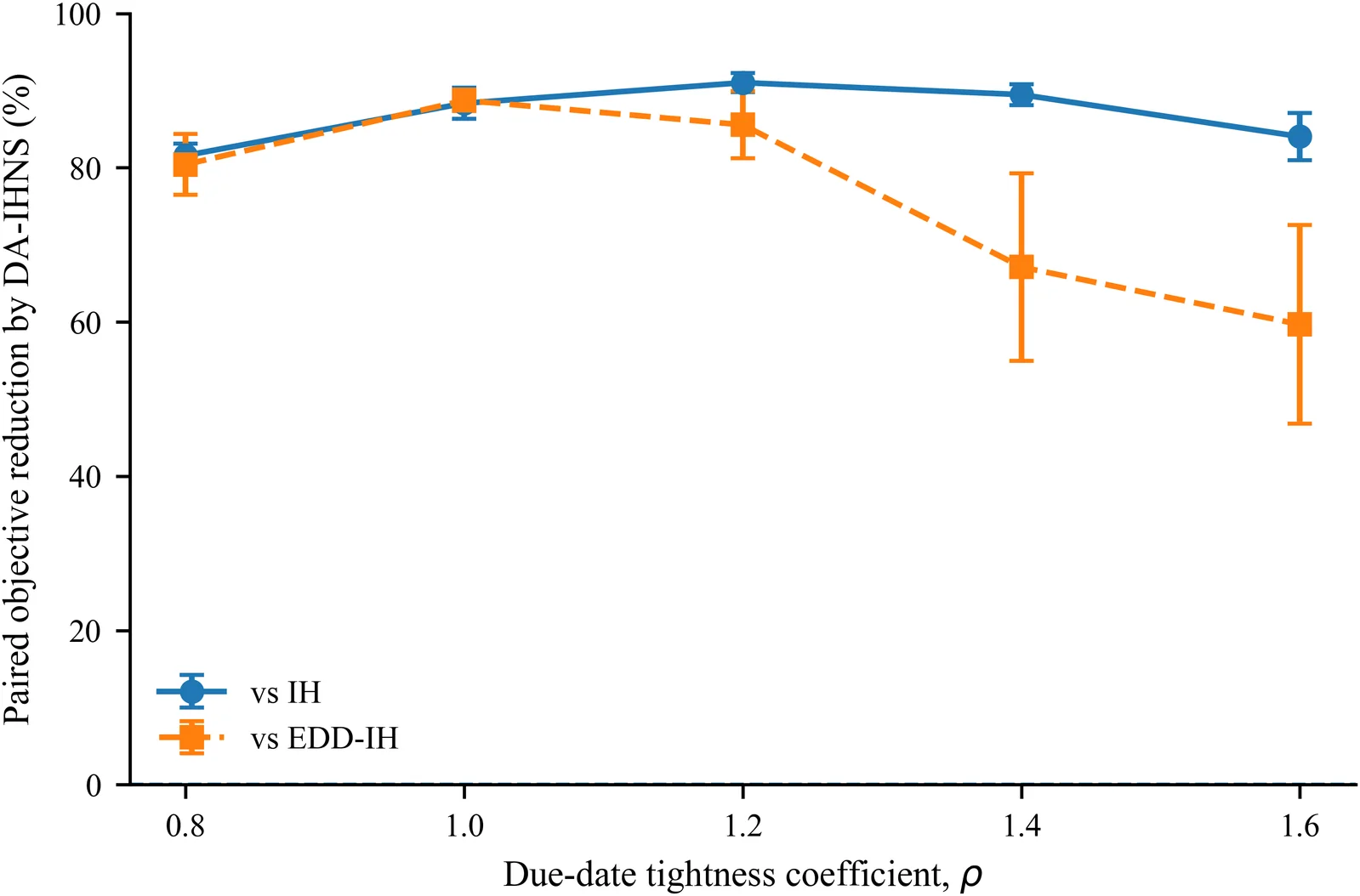
Paired objective reduction by DA-IHNS under different due-date tightness levels. Points show the mean paired reduction in the comprehensive objective value relative to IH and EDD-IH; error bars denote normal-approximation 95% confidence intervals for the mean.

### 5.6. Sensitivity to rack buffer capacity

The effect of rack buffer capacity is examined on the same 20 sensitivity instances with *N* = 50, *K* = 100, *S* = 150, and *C* = 8. Buffer capacity is varied over H∈{0,1,2,3,4,5,6,8,10}, while the order and rack data, due dates, algorithm parameters, and random seeds remain unchanged. The case *H* = 0 provides the no-buffer reference.

Mean rack retrievals are below the no-buffer level for all positive capacities tested. The larger reductions occur at low-to-moderate buffer capacities, whereas further increases in *H* do not produce systematic additional reductions. The results indicate diminishing additional reductions in rack retrievals as buffer capacity increases within the tested range. They do not imply that increasing buffer capacity necessarily improves the comprehensive objective.

### 5.7. Trade-off analysis of the tardiness penalty coefficient

The baseline experiments use λ=1. To examine the relative weighting of rack movements and weighted order tardiness, λ is varied over {0.25,0.5,1,2,4} while all other settings and the same 20 sensitivity instances are held fixed. The complete numerical results are reported in S3 Table.

As λ increases, mean weighted order tardiness decreases from 7.10 to 1.95, while mean rack movements increase from 14.95 to 18.70. The results show the trade-off between rack reuse and order timeliness. The value λ=1 is retained as a baseline scenario weight rather than a uniquely optimal or monetary coefficient.

## 6. Conclusions, managerial implications, and future research

### 6.1. Main conclusions

This paper studies the order and rack sequencing problem with a finite rack buffer area and order-specific due dates in a single-station RMFS. A mixed-integer programming model jointly determines the order entry sequence, rack service sequence, and buffer retention decisions to minimize rack movements, measured as rack retrievals from storage, and weighted order tardiness.

DA-IHNS combines normalized order urgency and rack coverage with order- and rack-level neighborhood search. In the exact-solution validation, DA-IHNS matches the CPLEX objective for all instances with *N* = 4 and 6 and shows only a small difference at *N* = 8; the deviation increases from *N* = 10 onward. In the larger paired experiments with *N* = 30, 50, and 80, DA-IHNS obtains lower comprehensive objective values than the two rule-based benchmarks, IH and EDD-IH, across the tested instances.

The additional experiments clarify the roles of the main operational and algorithmic components. Rack buffering primarily reduces repeated rack retrievals, while the rack-level neighborhood provides incremental intensification for a limited subset of order-search incumbents. The paired advantage over both rule-based benchmarks remains positive across the tested due-date tightness levels. Increasing buffer capacity yields diminishing additional reductions in rack retrievals, and variation in λ reveals the trade-off between rack reuse and order timeliness.

### 6.2. Managerial implications

Under the tested single-station settings, incorporating both order urgency and rack coverage can be preferable to relying on the tested due-date-only benchmark when rack reuse and order timeliness must be considered simultaneously.

Temporary rack retention reduces repeated rack retrievals from storage, with the larger reductions occurring at low-to-moderate buffer capacities in the tested instances. Further increases in buffer capacity provide limited additional reductions, indicating that the benefit of additional buffer positions depends on the rack-reuse opportunities available in the order mix.

The coefficient λ serves as a scenario weight for adjusting the relative emphasis on rack movements and weighted order tardiness. Larger values place greater emphasis on order timeliness, whereas smaller values favor rack reuse. Because λ is not calibrated from observed operating costs, it should not be interpreted as a monetary conversion factor.

### 6.3. Limitations and future research

The study is limited to a single picking station with all order information known at the beginning of the planning horizon. Rack operations are represented by rack-service time slots rather than rack-specific transportation times or robot congestion. The computational instances are synthetically generated, and the tardiness penalty coefficient λ is not calibrated from observed operating costs. The reported findings should therefore be interpreted within the tested computational settings.

Future research may extend the model to multiple picking stations, dynamic order arrivals, heterogeneous rack transportation times, and congestion-aware robot operations. Operational warehouse data could also be used to evaluate the model under application-specific order structures and to calibrate buffer-related and tardiness-related parameters.

## Supporting information

S1 Table Instance-level exact-solution validation results. Results for all exact-validation instances, including problem size, CPLEX objective value, best bound, solver status, MIP gap, DA-IHNS objective value, relative deviation, and CPU time.
(XLSX)

S2 TableOne-factor sensitivity analysis of the DA-IHNS scoring parameters.Results obtained by varying α, θ, δ, η, and ξ one at a time on the same 10 paired sensitivity instances while holding the remaining parameters at their baseline values.(XLSX)

S3 TableSensitivity analysis of the tardiness penalty coefficient.Results for λ∈{0.25,0.50,1.00,2.00,4.00} on the same 20 sensitivity instances, including the comprehensive objective value, rack movements, weighted order tardiness, and the corresponding summary statistics.(XLSX)

S4 Table Scale-level results of the main paired algorithm comparison. Mean comprehensive objective value, rack movements, weighted order tardiness, and CPU time for DA-IHNS, IH, and EDD-IH at *N* = 30, 50, and 80, based on the same 20 paired instances at each problem size.
(XLSX)

S5 Data Underlying computational data for all experiments. The workbook contains the instance metadata, complete instance-level results for the exact validation, main algorithm comparison, parameter sensitivity, tardiness-penalty sensitivity, buffer-capacity sensitivity, and due-date-tightness sensitivity experiments, together with the reported statistical test results.
(XLSX)
